# Transforming HIV Care: Adapting the Health4All Module to Combat the Stigma Among Malaysian Primary Healthcare Workers

**DOI:** 10.7759/cureus.69252

**Published:** 2024-09-12

**Authors:** Nor Fauziah Salaton, Rafdzah Ahmad Zaki, Sin How Lim, Natalia Che Ishak, Adeeba Kamarulzaman, Frederick Altice

**Affiliations:** 1 Social and Preventive Medicine, University Malaya, Kuala Lumpur, MYS; 2 Disease Control Division, Ministry of Health Malaysia, Putrajaya, MYS; 3 Internal Medicine, Monash University Malaysia, Subang Jaya, MYS; 4 Internal Medicine, Yale University, New Haven, USA

**Keywords:** health4all module, healthcare workers, hiv stigma, intervention, malaysia

## Abstract

Globally, stigma and discrimination in healthcare settings have been identified as significant barriers to implementing HIV prevention and treatment strategies. However, research on interventions to reduce the stigma in healthcare facilities, particularly in Malaysia, is lacking. Hence, this study aims to bridge these gaps by implementing a globally accepted training module to reduce HIV stigma among healthcare workers (HCWs). This study aims to implement the Health4All module to reduce HIV stigma among HCWs in Malaysia. A randomized controlled trial (RCT) study with repeated measures over a period of six months was conducted to implement the Health4All module and evaluate the study outcomes. The implementation process involved a total of 300 randomly selected HCWs from 10 primary health clinics in the study area. A total of 263 respondents participated at the beginning, giving a response rate of 88%, but only 177 completed records were used for the final analysis. The mean score for perceived risk and fear was reduced by 1.07 between baseline and one month (p < 0.001) and reduced further by 0.81 between one month and three months (p < 0.05). The mean score for value-driven stigma was reduced by 2.31 between baseline and one month (p < 0.001) and reduced further by 0.86 between one month and three months (p = 0.25). The mean score for discriminatory attitude was reduced by 1.65 between baseline and one month (p = 0.002) and reduced further by 1.65 between one month and three months (p = 0.002). The Health4All module was effective in reducing the HIV stigma among HCWs over three months. This study suggests that the training program can be replicated across different healthcare settings worldwide for wider implementation.

## Introduction

The acquired immunodeficiency syndrome (AIDS) epidemic continues to pose substantial challenges that require urgent action despite notable progress in available treatment. By the end of 2022, approximately 39 million people living with HIV (PLHIV) were reported globally [1]. Similarly, in Malaysia, the epidemic has been a major public health concern since the first case was identified in December 1986 [2]. As of late 2022, Malaysia had an estimated 86,142 PLHIV, with 69,589 (81%) having been informed of their status through the national surveillance system [3]. This ongoing situation highlights the need for sustained and innovative efforts to address the epidemic effectively.

Malaysia aims to eradicate AIDS by 2030 and has made notable progress over the past 20 years with a 65.8% decrease in the incidence of HIV from the year 2002 to the year 2022 [4]. Since 2000, HIV care and anonymous screening, which require no personal identification, have been available at all government health clinics, ensuring privacy and minimizing stigma [5]. However, achieving the 95-95-95 testing-treatment-viral suppression cascade remains a challenge, with only 68% of PLHIV receiving the treatment they need, highlighting a significant gap in reaching the goal [6]. Stigma and discrimination against PLHIV hinder efforts and remain a major barrier to controlling the epidemic [7,8]. Stigma is complex, with various definitions, but Goffman described it as a discrediting attribute that labels individuals as discreditable or dishonorable [9]. HIV-related stigma specifically targets PLHIV, manifesting as prejudice, discounting, discrediting, and discrimination [10].

Globally, stigma and discrimination against PLHIV are prevalent in healthcare settings, with the proportion of healthcare workers (HCWs) exhibiting stigmatizing attitudes varying significantly across different regions [11-13]. HCWs are crucial in fostering supportive environments and managing the disease [14]. However, negative attitudes and harmful behaviors from HCWs often deter PLHIV from seeking services, disclosing their health status, and adhering to treatment [15]. HCWs may either neglect to provide care or discourage treatment due to fear of stigma, which prevents PLHIV from receiving the necessary support [16]. Inadequate knowledge of HIV/AIDS correlates with increased discrimination against PLHIV, as insufficient understanding fosters fear of transmission and discriminatory attitudes, compounded by limited information dissemination and minimal community engagement [17-19].

Comprehensive HIV/AIDS training programs are vital for mitigating stigma and discrimination among HCWs and should be accessible to all HCWs, including those not directly involved in patient care [5,20,21]. Hence, this study adapted the Health4All module, a global training module developed by IntraHealth International in collaboration with the PEPFAR and USAID-funded LINKAGES project, a widely accessible resource available on their official website [22]. Since its introduction in 2016, this module has effectively trained 800 individuals from 14 countries worldwide with preliminary data showcased on the website indicating a positive impact on reducing HIV-related stigma in healthcare settings [23]. The Health4All module provides a distinctive approach that fosters supportive environments and enhances public health outcomes. Concurrently, the Ministry of Health Malaysia is committed to reducing HIV stigma among HCWs through a range of initiatives.

## Materials and methods

Study design and settings

This is an RCT with repeated measures, conducted from June 1, 2023 to December 31, 2023. This study was conducted involving 10 primary health clinics in three districts of Selangor, a state in Malaysia. The districts were selected based on the number of reported HIV cases. The study was conducted in two phases. The first phase involved the development of an intervention module. The second phase of the study involved an implementation of the intervention.

Eligibility criteria

Participants were eligible if they worked within the selected primary health clinics, aged between 18 and 65 years old, proficient or fluent in both English and Malay, and consented and willing to participate until the study is completed. Meanwhile, those who are digital illiterate, lacking proficiency in English and Malay, or with psychiatric illness were excluded from this study.

Recruitment of participants

The study targeted HCWs from 10 primary health clinics, with 300 participants randomly selected who fulfils the inclusion and exclusion criteria. The participants comprised all HCWs in clinic settings, including doctors, nurses, pharmacists, technicians, allied health professionals, administrative staff, and drivers, who collectively ensure comprehensive patient care. The recruitment process was conducted over a three-month period, during which all selected HCWs were thoroughly briefed and provided informed consent prior to their participation. Contact details were collected from those who agreed to participate in the study.

**Figure 1:**
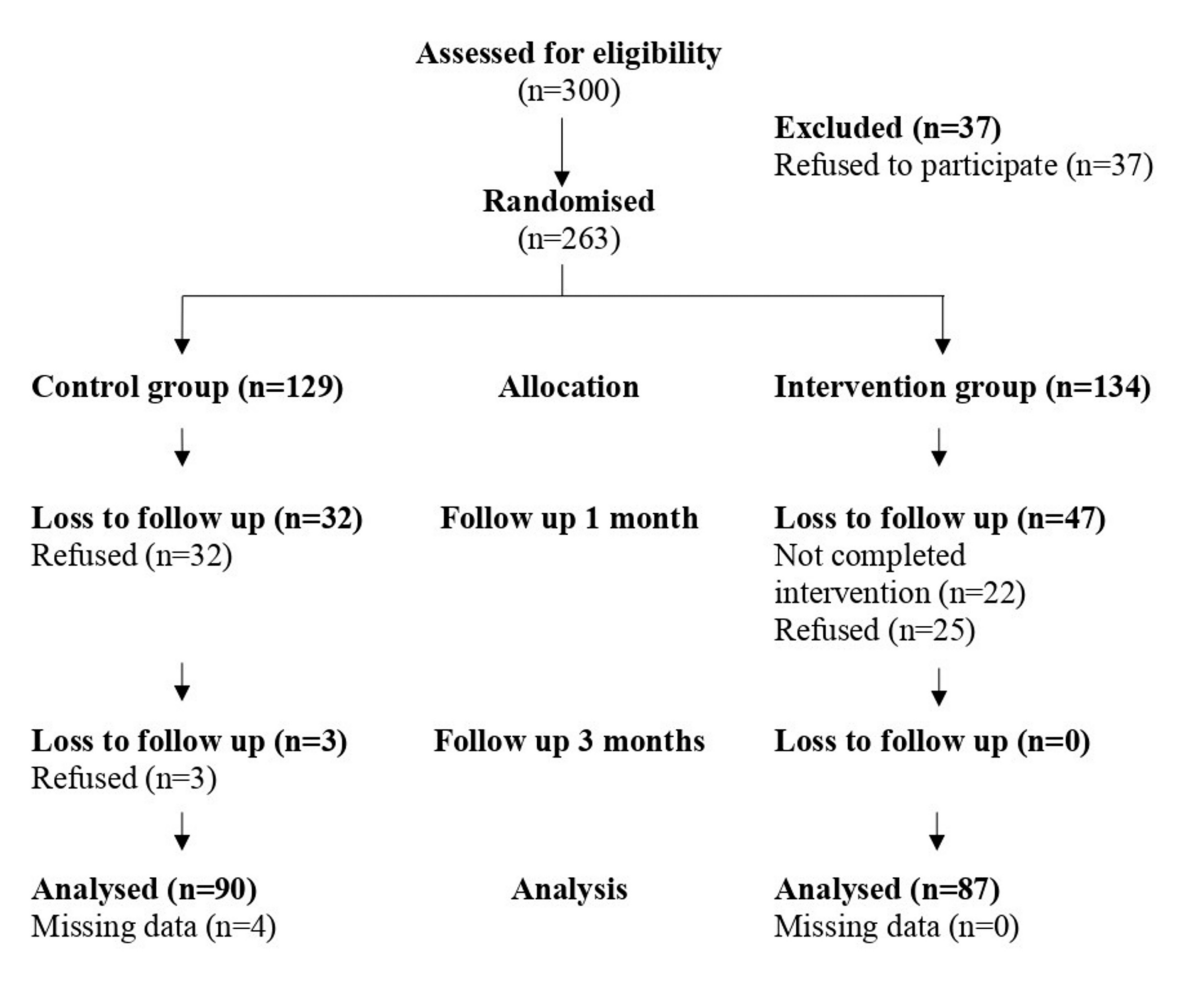
The CONSORT diagram

The flow of participants through each study stage, namely, enrollment, allocation, intervention exposure, and follow-ups, is depicted in Figure 1. Participants were assigned to the intervention and control groups in a 1:1 ratio, resulting in 150 HCWs in each group. Initially, 263 participants responded to the invitation and completed the baseline questionnaire (see Appendix), yielding an 88% response rate. The intervention group began with 129 participants, while the control group started with 134 participants. In the intervention group, 64% (87 participants) completed the intervention, with non-completion mainly due to withdrawal or refusal, as those who withdrew did not respond to follow-up, possibly due to tight working schedules. At the one-month follow-up, 35 participants from the control group and 46 from the intervention group were lost to follow-up. At the three-month follow-up, one participant from the control group was lost to follow-up, with none lost from the intervention group. By the end of the study, 177 records were available for analysis, considering 82 participants lost to follow-up and four records with missing data.

Sample size calculation

The sample size calculation was based on the results by Pulerwitz et al. [24]. The values were entered into the G*Power version 3.1.9.6 program to calculate the sample size for repeated-measure ANOVA with a power of 80% and significant level preset at 0.05 and effect size of 0.44. Assuming a drop-out rate of 50% in view of an online study and to ensure adequate power was established for most of the outcome measures, the total number of participants required for this study was 300 (150 for each group).

Intervention

This study adapted the publicly accessible Health4All module, a global training program to reduce HIV stigma in healthcare settings as illustrated in Table 1. It includes education on stigma, the biopsychosocial impact of HIV, and empathy-building sessions with PLHIV. The development of the intervention involved adapting the original module content and translating it into an online format due to the COVID-19 pandemic and the impracticality of conducting in-person workshops in busy clinic environments. First, the adaptation process was executed by experts from the Ministry of Health Malaysia, who have extensive experience in HIV/AIDS. They tailored the module to fit the local context and health needs by integrating the epidemiology of HIV in Malaysia and addressing unique issues and challenges in managing PLHIV in local settings. The adaptation process produced a series of PowerPoint slides with accompanying narrator scripts.

**Table 1:** The title and objectives of modules in the Health4All module

Title of the modules	Objectives of the modules
Module 1: The Rationale for Services for Key Populations	
Session 1.1: Know your epidemic. Session 1.2: Why focus on key populations? Session 1.3: Gender and social norms. Session 1.4: Substance use. Session 1.5: Violence, key populations, and human rights	Introduces participants to key populations and explains why providing quality and comprehensive services for key populations is critically important in the response to HIV.
Module 2: Hearts and Minds: Quality Services for Key Populations	
Session 2.1: Beliefs about key populations. Session 2.2: Our values, judgments, and opportunities to challenge stigma. Session 2.3: Forms, causes, layers, and effects of stigma	This module encourages participants to recognize and confront stigma and discrimination.
Module 3: Appropriate Services for Key Populations	
Session 3.1: Top 10 clinical standards of care for key populations. Session 3.2: Providing youth-friendly services to key populations. Session 3.3: Performing a risk assessment. Session 3.4: Suggested session: Panel discussion	This module reviews the priority clinical standards of care that are tailored to meet the unique needs of key population
Module 4: Action, Change, Commitment	
Session 4.1: Monitoring service quality using LINK. Session 4.2: Creating a key-population-friendly clinic. Session 4.3: Planning for action in your health facility.	This module consolidates the commitments that are required of providers, and facilities to address stigma & discrimination in health care settings.

Subsequently, the training module was transitioned to an online platform to ensure accessibility and flexibility, allowing participants to engage with the content at their convenience (https://health4alltraining.wixsite.com/health4all). The online platform development involved finalizing the module content, converting it into multiple short videos, and enhancing engagement with animations and infographics. The PowerPoint presentations were transformed into a video format with voice-over in Malay to accommodate HCWs with limited English proficiency.

To further enrich the training, two pre-recorded videos were embedded on the website. These videos featured sharing sessions from PLHIV and representatives from non-governmental organizations (NGOs) actively involved in HIV prevention in Malaysia. Permissions were secured for these recordings, and the confidentiality of PLHIV was maintained. The PLHIV shared their personal experiences with stigma and discrimination in healthcare settings, while the NGO representatives discussed their contributions to managing the psychosocial aspects, including financial support, for PLHIV in Malaysia.

In lieu of the original module’s interactive sessions, the online training included a discussion area for participants to share their experiences and opinions on stigma-related issues through open-ended questions. Participants submitted their responses via a provided link, and the researcher was available for queries through an embedded contact link. This approach not only aimed to enhance participants' knowledge but also facilitated progress monitoring, with follow-up reminders issued to those demonstrating poor progress.

The intervention for participants started on July 1, 2023, and concluded on July 31, 2023, spanning one month. Meanwhile, the control group was not undergoing the current existing intervention and not receiving any intervention. After assessing their baseline HIV stigma and discrimination, the participants in the intervention group were provided with a link to the website domain. The asynchronous learning approach required participants to complete the online training module at their own pace, ensuring they could do so without interfering with their work hours or clinic operations. To begin the intervention, participants create an account on the website using a unique ID and login for each visit. After completing each module, they submit feedback through an integrated system. Researchers receive automatic email notifications of this feedback and can remind participants who miss deadlines. These measures effectively monitor participants' progress and activities.

Outcome measure

To evaluate the effectiveness of the Health4All module in mitigating HIV-related stigma and discrimination among participants, three outcome measures were measured: perceived risk and fear of HIV/AIDS, value-based stigma, and discriminatory attitudes toward individuals with HIV/AIDS. Measurements were taken at baseline, one month, and three months after completing the module, using the same set of validated questionnaires, for which permission was obtained from the author [25]. The author is a local scholar who has completed her doctoral studies, concentrating on evaluating HIV stigma and discrimination among healthcare personnel in Malaysia, with the aim of deepening understanding and addressing these critical issues.

Perceived risk and fears toward HIV/AIDS

Perceived risk and fear toward HIV/AIDS are defined as HCWs’ feeling unsafe and precarious and having fear of HIV transmission during various types of contact and medical procedures with HIV-positive patients. There are seven items in this variable, and it is measured using a four-point Likert scale [25]. “Never considered as risk” is scored as “1," “No risk and fear” is scored as “2,” “Moderate risk and has fear” is scored as “3,” and “High risk and fearful” is scored as “4.” Scores one and two show that the HCWs has not perceived risk and fear toward HIV/AIDS, and scores three and four represent perceived risk and fear while handling patients with HIV/AIDS. The minimum total score for the variable perceived risk and fear is 7, and the maximum total score is 28. For the analysis, the total score of 7 to 14 is implied as not perceived risk and fear, and the total score of 15 to 28 is implied as perceived risk and fear toward HIV/AIDS.

Value-driven stigma

Value-driven stigma is defined as ideas or viewpoints of HCW that are associated with shaming, blaming, and being judgemental toward PLHIV. Overall, there are 23 items in this variable [25]. Its four-point Likert scale scored “Strongly disagree” is scored “1," “Disagree” scored “2," “Agree” scored “3,” and “Strongly agree” scored “4." Scores one and two show that HCWs do not have any value-driven stigma toward HIV and scores three and four shows to have a value-driven stigma toward the disease. Since there are 23 items in this variable, the minimum total score for this variable is 23 and the maximum total score is 92. For the analysis, the total score of 23 to 46 is implied as not having any value-driven stigma, and the total score of 47 to 92 is implied as having value-driven stigma toward HIV/AIDS.

Discriminatory attitude toward HIV/AIDS

A discriminatory attitude toward HIV/AIDS is defined as prejudice, harsh, or poor behavior by HCWs toward HIV-infected individuals. The performance of the actual belief of stigma results in a discriminatory attitude towards the disease. There are 15 items (questions) in this variable [25]. It is measured using a four-point Likert scale. “Strongly disagree” is scored as “1," “Disagree” scored as “2," “Agree” scored as “3,” and “Strongly agree” is scored as “4." Scores one and two show that HCWs disagree the behavior of discrimination, and scores three and four show that HCWs agree to have a discriminatory attitude toward the illness. Since there are 15 items in this variable, the minimum total score for this variable is 15 and the maximum total score is 60. For the analysis of this study, the total score of 15 to 30 is implied as having no discriminatory attitude towards HIV/AIDS, and the total score of 31 to 60 is implied as having discrimination toward HIV/AIDS.

Statistical analysis

A descriptive analysis of the most representative independent variables following the sociodemographic characteristics and HIV-related descriptions was done. Categorical data were expressed as absolute numbers (n) and percentages (%), while continuous data were expressed as mean (𝑥̅) and standard deviation (SD). To evaluate the effectiveness of the intervention, a repeated-measure ANOVA test was carried out. This allowed the researcher to compare the mean between two groups (intervention and control) from three different measurement periods (pre-training, post-training for one month, and post-training for three months). The analysis was performed according to the per-protocol principle. The assumptions were checked before proceeding with this analytical test. To check for data normality, the dataset was tested for the presence of skewness and kurtosis. Subsequently, a Mauchly test was carried out to check for the assumption of sphericity. A significant result greater than 0.05 indicates that the assumption for sphericity is not violated, so the dataset can be used for the repeated-measure ANOVA test. The result from the repeated-measure ANOVA was expressed using the mean difference (MD) and standard error (SE) following adjustment using the Bonferroni method. IBM SPSS Statistics for Windows, Version 26.0 (IBM Corp., Armonk, NY) was used for all analyses.

Ethical approval and consent

This study was conducted following the ethical principles outlined in the Declaration of Helsinki and the Malaysian Good Clinical Practice Guideline. The protocol of this study has obtained ethical clearance from the Ministry of Health’s Medical Research and Ethics Committee (MREC) and National Medical Research Registry (NMRR) - NMRR ID-22-01643-DZT (IIR). This study was also approved by the Selangor State Health Department before commencement. Written informed consent was obtained from all participants before the study began.

## Results

Baseline characteristics of the participants

A total of 263 respondents participated at the outset, achieving a response rate of 88%. However, only 259 respondents completed the baseline questionnaire. The sociodemographic and occupational characteristics of these respondents are presented in Table 2. Most respondents were female (195 (75.3%)) with an average age of 35.5 ± 5.96 years. The majority were Malay (206 (79.5%)) and Muslim (211 (81.5%)). More than half were married (206 (79.5%)). Of all the respondents, 221 (85.3%) were from the clinical group, with most having less than 15 years of work experience (214 (82.6%)). The majority were not working in an HIV clinic (184 (71.0%)), about half had experience treating PLHIV (151 (58.3%)), and most had never attended any training related to HIV/AIDS (192 (74.1%)).

**Table 2:** Sociodemographic characteristic of the participants at baseline, N = 259 *Missing one value, ** missing two values, ^a^Chi-Square test. The p-value is set as significant at <0.05.

Variables	Items	Total	Study arm’s group		p-value^a^
			Control	Intervention	
Age category* (Mean 35.5 ± 5.96)	Less than 40 years old	213 (82.2)	106 (82.2)	108 (83.1)	0.85
	41 years old and more	45 (17.4)	23 (17.8)	22 (16.9)	
Gender	Man	64 (24.7)	33 (25.6)	31 (23.8)	0.75
	Woman	195 (75.3)	96 (74.4)	99 (76.2)	
Ethnicity*	Malay	206 (79.5)	100 (77.5)	107 (82.3)	0.34
	Not Malay	52 (20.1)	29 (22.5)	23 (17.7)	
Religion*	Muslim	211 (81.5)	107 (82.9)	105 (80.8)	0.65
	Non-Muslim	47 (18.1)	22 (17.1)	25 (19.2)	
Marital status*	Married	206 (79.5)	100 (77.5)	107 (82.3)	0.34
	Not married	52 (20.1)	29 (22.5)	23 (17.7)	
Professional designation*	Clinical	221 (85.3)	108 (83.7)	117 (90.0)	0.14
	Non-clinical	37 (14.3)	21 (16.3)	13 (10.0)	
Number of working years**	Less than 15 years	214 (82.6)	105 (81.4)	110 (84.6)	0.49
	16 years and more	43 (16.6)	24 (18.6)	20 (15.4)	
Experience in treating HIV patients*	Yes	151 (58.3)	68 (52.7)	83 (63.8)	0.07
	No	107 (41.3)	61 (47.3)	47 (36.2)	
Working in an HIV speciality clinic	Yes	75 (29.0)	27 (20.9)	48 (36.9)	0.05
	No	184 (71.0)	102 (79.1)	82 (63.1)	
Receive training or courses related to HIV/AIDS in the past one year	Yes	67 (25.9)	27 (20.9)	40 (30.8)	0.07
	No	192 (74.1)	102 (79.1)	90 (69.2)	

Descriptive analysis of perceived risk and fear, value-driven stigma, and discriminatory attitude among the participants

The descriptive analysis of perceived risk and fear, value-driven stigma, and discriminatory attitude among the participants are detailed in Table 3. The majority, 89%, reported perceived risk and fears toward HIV/AIDS, primarily among those under 40 years old (196, 84.1%), female (175, 75.1%), married (186, 79.8%), Malay (186, 79.8%), and Muslim (194, 83.3%). Occupationally, they were mostly from the intervention group (117, 50.2%), clinical jobs (200, 85.8%), had experience treating PLHIV (134, 57.5%), less than 15 years of work experience (197, 84.5%), did not work in HIV clinics (173, 74.2%), and had not received HIV/AIDS training (177, 76.0%).

**Table 3:** Perceived risk and fear, value-driven stigma and discriminatory attitude among participants at baseline, N = 259 *Missing one value, **missing two values

Variables	Items	Frequency, N (%)			
		Total	Perceived risk and fear, 233 (89)	Value-driven stigma, 200 (77)	Discriminatory attitude, 105 (41)
Age category* (Mean 35.5 ± 5.96)	Less than 40 years old	213 (82.2)	196 (84.1)	165 (82.5)	81 (77.1)
	41 years old and more	45 (17.4)	37 (15.9)	35 (17.5)	24 (22.9)
Gender	Man	64 (24.7)	58 (24.9)	49 (24.4)	22 (21.0)
	Woman	195 (75.3)	175 (75.1)	152 (75.6)	83 (79.0)
Ethnicity*	Malay	206 (79.5)	186 (79.8)	162 (81.0)	84 (80.0)
	Not Malay	52 (20.1)	47 (20.2)	38 (19.0)	21 (20.0)
Religion*	Muslim	211 (81.5)	194 (83.3)	176 (88.0)	99 (94.3)
	Non-Muslim	47 (18.1)	39 (16.7)	24 (12.0)	6 (5.7)
Marital status*	Married	206 (79.5)	186 (79.8)	162 (81.0)	84 (80.0)
	Not married	52 (20.1)	47 (20.2)	38 (19.0)	21 (20.0)
Group of health clinic	Control	129 (49.8)	116 (49.8)	107 (53.2)	57 (54.3)
	Intervention	130 (50.1)	117 (50.2)	94 (46.8)	48 (45.7)
Professional designation*	Clinical	221 (85.3)	200 (85.8)	164 (82.0)	81 (77.1)
	Non-clinical	37 (14.3)	33 (14.2)	36 (18.0)	24 (24.0)
Number of working years**	Less than 15 years	214 (82.6)	197 (84.5)	163 (81.9)	78 (74.3)
	16 years and more	43 (16.6)	36 (15.50)	36 (18.1)	27 (25.7)
Experience in treating HIV patient*	Yes	151 (58.3)	134 (57.5)	100 (50.0)	42 (40.0)
	No	107 (41.3)	99 (42.5)	100 (50.0)	63 (62.0)
Working in an HIV speciality clinic	Yes	75 (29.0)	60 (25.8)	49 (24.4)	23 (21.9)
	No	184 (71.0)	173 (74.2)	152 (75.6)	82 (78.1)
Receive training or course related to HIV/AIDS in the past one year	Yes	67 (25.9)	56 (24.0)	40 (19.9)	15 (14.3)
	No	192 (74.1)	177 (76.0)	161 (80.1)	90 (85.7)

Regarding value-driven stigma, 77% of the respondents reported this sentiment, mainly among those under 40 (165, 82.5%), female (152, 75.6%), married (162, 81.0%), Malay (162, 81.0%), and Muslim (176, 88.0%). Occupational characteristics included a majority from the control group (107, 53.2%), clinical jobs (164, 82.0%), experience treating PLHIV (100, 50.0%), less than 15 years of work experience (163, 81.9%), not working in HIV clinics (152, 75.6%), and no previous HIV/AIDS training (161, 80.1%).

A discriminatory attitude toward HIV/AIDS was reported by 41% of the participants, predominantly among those under 40 (81, 77.1%), female (83, 79.0%), married (84, 80.0%), Malay (84, 80.0%), and Muslim (99, 94.3%). Occupationally, they were mostly from the control group (57, 54.3%), clinical jobs (81, 77.1%), had experience treating PLHIV (63, 62.0%), less than 15 years of work experience (78, 74.3%), did not work in HIV clinics (82, 78.1%), and had not received HIV/AIDS training (90, 85.7%).

Perceived risk and fears, value-driven stigma, and discriminatory attitude

A repeated-measure ANOVA was performed to evaluate the effect of time and group (independent variables) on outcomes - perceived risk and fear, value-driven stigma, and discriminatory attitude (dependent variables) among the participants. 

Perceived risk and fears

The normality and sphericity of the data were assumed, and the degrees of freedom were not corrected. The Mauchly's test was significant, χ2(2) = 1.67, p = 0.434. The repeated measures ANOVA revealed a significant main effect of time, F (2, 350) = 17.052, p < 0.01, ηp² = 0.089. As the main ANOVA is significant, this means that there is a difference between at least two time points.

The pairwise comparisons were then carried out with a Bonferroni correction to keep the type 1 error at 5% overall, and the result is shown in Table 4. There is a significant reduction in the mean score for the intervention group as the CI for post one month and post three months do not overlap with the baseline. Meanwhile, the mean difference for perceived risk and fear across time points and groups is shown in Table 5. Perceived risk and fear reduced by 1.07 between baseline and one month (95% CI: 0.28-1.86, p < 0.001) and then reduced by an additional 0.81 between one month and three months (95% CI: 0.07-1.56, p < 0.05), indicating a significant and meaningful reduction.

**Table 4:** Repeated-measure ANOVA test and pairwise comparison of the study outcomes across time points.

Outcomes	Group	Time	Mean	SE	95% CI
Perceived risk and fears	Control	Baseline	20.33	0.37	19.60–21.06
		Post one month	20.49	0.50	19.49–21.49
		Post three months	19.90	0.47	18.97–20.84
	Intervention	Baseline	18.63	0.39	17.85–19.40
		Post one month	16.33	0.54	15.26–17.39
		Post three months	15.29	0.50	14.29–16.28
Value-driven stigma	Control	Baseline	54.89	0.92	53.08–56.08
		Post one month	54.04	1.04	51.98–56.10
		Post three months	53.22	1.14	50.97–55.48
	Intervention	Baseline	49.82	0.98	47.89–51.75
		Post one month	46.06	1.11	43.87–48.25
		Post three months	45.17	1.22	42.77–47.57
Discriminatory attitude	Control	Baseline	27.67	0.70	26.28–29.06
		Post one month	26.42	0.83	24.78–28.05
		Post three months	27.68	0.79	26.11–29.26
	Intervention	Baseline	25.65	0.75	24.17–27.13
		Post one month	23.61	0.88	21.87–25.36
		Post three months	22.00	0.85	20.32–23.68

**Table 5:** Repeated-measure ANOVA test and pairwise comparison of the mean difference for outcomes across groups and times. *Repeated-measure ANOVA test. The p-value is set as significant at <0.05.

Outcomes	Time (I)	Time (J)	Mean difference (I-J)	SE	P value*	95% CI
Perceived risk and fear	Baseline	One month	1.07	0.33	<0.01	0.28-1.86
	One month	Three months	0.81	0.31	0.03	0.07-1.56
Value-driven stigma	Baseline	One month	2.31	0.54	<0.01	1.01-3.60
	One month	Three months	0.86	0.49	0.25	0.33-2.04
Discriminatory attitude	Baseline	One month	1.65	0.46	<0.01	0.52-2.77
	One month	Three months	1.65	0.46	<0.01	2.77-0.52

Value-driven stigma

The normality and sphericity of the data were assumed, and the degrees of freedom were not corrected. The Mauchly's test was significant, χ2(2) = 4.89, p = 0.087. The repeated-measure ANOVA revealed a significant main effect of time, F (1, 175) = 30.95, p < 0.01, ηp² = 0.15. As the main ANOVA is significant, this means that there is a difference between at least two time points.

The pairwise comparisons were then carried out with a Bonferroni correction to keep the type 1 error at 5% overall, and the result is shown in Table 4. There is a significant reduction in the mean score for the intervention group. Meanwhile, the mean difference for value-driven stigma across time points and groups is shown in Table 5. Value-driven stigma was significantly reduced by 2.31 between baseline and one month (95% CI: 1.01-3.60, p < 0.001) and then further reduced by an additional 0.86 between one month and three months even though it was not significant (95% CI: 0.33-2.04, p = 0.25).

Discriminatory attitude

The normality and sphericity of the data were assumed, and the degrees of freedom were not corrected. The Mauchly's test was significant, χ2(2) = 2.624, p = 0.269. The repeated-measure ANOVA revealed a significant main effect of time, F (2, 350) = 8.89, p < 0.01, ηp² = 0.048. As the main ANOVA is significant, this means that there is a difference between at least two time points.

The pairwise comparisons were then carried out with a Bonferroni correction to keep the type 1 error at 5% overall, and the result is shown in Table 4. There is a significant reduction in the mean score for the intervention group. Meanwhile, the mean difference for discriminatory attitudes across time points and groups is shown in Table 5. Discriminatory attitude reduced by 1.65 between baseline and one month (95% CI: 0.52-2.77, p = 0.002) and then reduced by an additional 1.65 between one month and three months (95% CI: 2.77-0.52, p = 0.002) indicating a significant and meaningful reduction.

## Discussion

This study found that the participants still harbored stigma and discrimination despite advances in HIV treatment, with 89.9% perceiving risk and fear, 77% exhibiting value-driven stigma, and 40.5% showing discriminatory attitudes toward PLHIV. Most participants, working in HIV-specialized clinics, had not received recent HIV-related training, highlighting the need for comprehensive stigma reduction programs that provide comprehensive education on HIV transmission, prevention, and treatment and dispel myths and misconceptions. This stigma reduction program could employ a variety of methods, integrating both theoretical and practical approaches. It would not only focus on educational training but also include role-playing, group discussions, and interactions with representatives of PLHIV [13, 26].

The Health4All module is designed to be inclusive and accessible to HCWs with diverse educational backgrounds, presenting content clearly and catering to various levels of expertise. This study demonstrates the effectiveness of the Health4All module in reducing HIV stigma and discrimination among HCWs, offering flexibility and accessibility tailored to busy schedules. These findings are congruent with other global studies targeting to reduce HIV stigma in healthcare settings and provide evidence that stigma reduction interventions can be effective. Regardless of the HIV prevalence settings of the respective countries, educational intervention provides impactful findings and benefits HCWs in terms of reducing their HIV stigma and discrimination [20,27].

The Health4All module effectively dispelled misconceptions about HIV transmission and treatment, providing HCWs with accurate information and a broader understanding of HIV/AIDS' societal and public health implications. This training not only improved HCWs' knowledge but also promoted key population-friendly health services, fostering a compassionate and inclusive healthcare system that benefits PLHIV and aids in combating the epidemic. In addition, this module addresses the root causes of stigma by promoting cultural competency and diversity awareness and educating HCWs about the unique challenges faced by PLHIV to foster a more inclusive healthcare environment [28]. Its effectiveness is enhanced by incorporating real-life stories from PLHIV, providing practical insights into empathetic patient care, and encouraging HCWs to approach PLHIV with sensitivity, with studies showing that such collaboration amplifies the impact of interventions [24,29].

The Health4All module is well-suited for reducing HIV stigma among HCWs in busy clinic settings by providing concise and accessible information that dispels misconceptions and fosters a nuanced understanding of HIV. Its emphasis on confidentiality, open communication, and empathy, combined with the flexibility of online learning, allows HCWs to engage with the content at their own pace, making it particularly effective in the fast-paced environment of clinical settings. In addition, this study utilized an online platform to deliver the Health4All module, offering flexibility for HCWs and facilitating ongoing learning, like a successful online intervention in India [20]. Despite the shift from in-person to online training due to the COVID-19 pandemic, learner outcomes were not significantly affected, and the online format enabled large-scale training while accommodating the diverse needs of HCWs.

Strengths and limitations of the study

This study is the first in Malaysia to implement the globally recognized Health4All training module to reduce HIV stigma among HCWs in Selangor clinics, demonstrating a significant strength of the research. The module provides a systematic approach to delivering consistent, evidence-based information and is adaptable to local healthcare issues, promoting a stigma-free environment. The RCT design enhances internal validity and establishes causal relationships by randomly assigning participants to intervention and control groups, thus reducing selection bias and confounding variables. As the gold standard for evaluating intervention effectiveness, RCTs provide a robust foundation for evidence-based decision-making in various fields, including social sciences and medicine.

Despite the rigorous methodology of the RCT study design, it has significant limitations, including a potential lack of generalizability and high dropout rates that can affect the validity and reliability of findings. With a 33% dropout rate in this study, bias may be introduced, and strategies such as examining dropout reasons and adjusting the analysis are crucial, alongside considering both the intention to treat (ITT) and per protocol (PP) analysis, with ITT generally being superior for maintaining validity.

The online platform is advantageous for providing continuous education to HCWs by allowing flexible access to training modules and accommodating their busy schedules, while also being scalable to reach a diverse audience. However, challenges such as low participant engagement, the impersonal nature of digital platforms, and varying levels of digital literacy may lead to high dropout rates, which can be mitigated by designing engaging content, considering higher nonresponse rates in sample size calculations, and ensuring a user-friendly website.

Time constraints posed a significant limitation for HCWs in this study, as balancing patient care with completing online modules led to procrastination or abandonment of the training. However, a robust monitoring system with frequent follow-up reminders mitigated the dropout rate by providing real-time tracking of progress and participation, offering tailored support, and accommodating HCWs' busy schedules.

## Conclusions

The implementation of the Health4All module to reduce HIV stigma in healthcare settings has emerged as a resounding success. This study yields essential findings that will aid policymakers in refining training and educational programs for HCWs. By providing HCWs with up-to-date medical knowledge, dispelling myths, and fostering cultural competency, the module addressed the root causes of stigma comprehensively. This achievement not only benefits PLHIV but also enriches the overall quality of care and inclusivity within healthcare settings. The Health4All module not only succeeded in imparting knowledge but also catalyzed a profound shift in attitudes and behaviors. Its success is a testament to its holistic approach, addressing the multifaceted aspects of stigma within the healthcare context. This accomplishment not only benefits those affected by HIV but sets a precedent for more compassionate, equitable, and stigma-free healthcare practices, marking a significant milestone in fostering a culture of understanding and inclusivity. Tackling and eliminating stigma within healthcare settings is critical for progressing towards our country's ambitious goal of ending the AIDS epidemic by 2030.

## Appendices

Questionnaire measuring study outcomes

Title: Transforming HIV Care: Adapting the Health4All Module to Combat Stigma Among Malaysian Primary Healthcare Workers

Respected respondents,

This questionnaire is divided into four sections: demographic, perceived risk and fear, value-driven stigma and discriminatory attitudes. We, therefore, request you your cooperation in answering the enclosed research questions. Your feedback is crucial in assessing how well this module addresses stigma-related issues. Any additional feedback or suggestions are greatly appreciated.

The information will be kept strictly confidential

Section One: Demographic Information

Please fill in and tick (√) for an appropriate answer.

**Table 6:** Demographic information

No	Question
101.	a.What are the first two initials of your first (given) name? Do not state your full name (Example: If your name is Mohd Abu Bakar bin Khalid Ibrahim = MA)
	b. What are the first two initials of your mother's first (given) name? Do not state full name (Example: Sharifah Siti Maimunah binti Syed Ali = SS)
102.	Date of birth: _____ (day/month/year)
103.	Age: _____(years)
104.	Sex: Male Female
105.	Marital status: Married Divorced Widowed Never married/Single
106.	Ethnic group: Malay Chinese Indian Others. If others, please specify ___
107.	Religion: Muslim Buddhist Hindu Christian Others. If others, please specify __
108.	In your career experience, have you cared for or treated people living with HIV? Yes No I don’t know
109.	What is your professional designation? Specialist Medical Officer Nurse Attendant Pharmacist Lab technician Registration staff Administration staff Security guard Driver
110.	How many years have you been in the involved in your work field? Please state number of years in service: _____
111.	Do you currently work in an HIV speciality Clinic? Yes No
112.	Did you receive training or courses related to HIV/AIDS past one year? Yes No
113.	How many times of training or courses related to HIV/AIDS did you attended past one year?

Section Two: Perceived Risk and Fear

We kindly request that you adopt a neutral approach in your answers and use the following rating scale:

1.     High risk

2.     Moderate risk

3.     No risk

4.     Never consider a risk

**Table 7:** Perceived risk and fear

Questions
201. Touching the sweat of a person with HIV or AIDS 1 2 3 4
202. Touching the saliva of a person with HIV or AIDS 1 2 3 4
203. Giving an injection to a person with HIV or AIDS 1 2 3 4
204. Caring for a person with HIV 1 2 3 4
205. Dressing the wounds of a person with HIV or AIDS 1 2 3 4
206. Conducting surgery on or suturing a person with HIV or AIDS 1 2 3 4
207. Putting an intravenous drip in someone who is showing signs of AIDS 1 2 3 4

Section Three: Value-Driven Stigma

We kindly request that you adopt a neutral approach in your answers and use the following rating scale:

1.     Strongly agree

2.     Agree

3.     Disagree

4.     Strongly disagree

**Table 8:** Value-driven stigma

Questions
301. I avoid touching the clothing and belongings of clients known or suspected to have HIV for fear of becoming HIV-infected 1 2 3 4
302. The most frequent mode of contracting HIV among health care providers is through work-related exposure 1 2 3 4 .
303. The most frequent mode of contracting HIV among health care providers get infected at work 1 2 3 4
304. HIV is a punishment from God 1 2 3 4
305. HIV is punishment for bad behavior 1 2 3 4
306. People with HIV should be ashamed of themselves 1 2 3 4
307. Promiscuous men spread HIV in our community 1 2 3 4
308. Female prostitutes spread HIV 1 2 3 4
309. I would feel ashamed if I was infected with HIV 1 2 3 4
310. I would feel ashamed if someone in my family was infected with HIV 1 2 3 4
311. I would prefer not to provide services to - People who inject illegal drugs 1 2 3 4
312. I would prefer not to provide services to - Men who have sex with men 1 2 3 4
313. I would prefer not to provide services to - Sex workers 1 2 3 4
314. I would prefer not to provide services to - Transgender people 1 2 3 4
315. I would prefer not to provide services to - Women who have sex with women 1 2 3 4
316. I would prefer not to provide services to - Migrants 1 2 3 4
317. If a pregnant woman is HIV positive, her family has a right to know 1 2 3 4
318. Pregnant women who refuse HIV testing are irresponsible 1 2 3 4
319. Women living with HIV are unable to be good mothers 1 2 3 4
320. Women living with HIV who do not follow infant feeding recommendations for preventing transmission of HIV to their infants are irresponsible 1 2 3 4
321. Women living with HIV should not get pregnant if they already have children 1 2 3 4
322. A pregnant woman living with HIV should undergo antiretroviral therapy, even if this is not her choice, for the health of the baby 1 2 3 4
323. It can be appropriate to sterilize a woman living with HIV, even if this is not her choice 1 2 3 4

Section Four: Discriminatory Attitudes Toward HIV/AIDS

We kindly request that you adopt a neutral approach in your answers and use the following rating scale:

1.     Strongly agree

2.     Agree

3.     Disagree

4.     Strongly disagree

**Table 9:** Discriminatory attitudes toward HIV/AIDS

Questions
401. Those who have HIV and AIDS should not be allowed to mix freely with other people 1 2 3 4
402. I prefer to refer persons with HIV and AIDS to other physicians/care providers 1 2 3 4
403. I do not want persons at high risk for HIV and AIDS as my patients 1 2 3 4
404. Children with HIV and AIDS should not be allowed to attend public schools 1 2 3 4
405. If I had a choice, I would not work with HIV/AIDS patients 1 2 3 4
406. People who have HIV and AIDS should not be allowed to work 1 2 3 4
407. Those who have HIV and AIDS should be forced to resign from their job 1 2 3 4
408. Needs of people with HIV should not be given priority 1 2 3 4
409. I would request my authority to remove me from the responsibility of caring HIV patients 1 2 3 4
410. Medical students who are HIV-positive should not have the right to complete their degree 1 2 3 4
411. Government should spend less money on HIV and AIDS, and more on other, more common diseases 1 2 3 4
412. Our country does not need any laws to protect people with HIV and AIDS from discrimination 1 2 3 4
413. I do not have responsibility to treat people with HIV and AIDS 1 2 3 4
414. I am not comfortable assisting or being assited by a colleague who is HIV-infected 1 2 3 4
415. I am not comfortable performing surgical or invasive procedures on clients whose HIV status is unknown 1 2 3 4
